# Clinicopathological analysis and genomic profiling of a rare histiocyte-rich rhabdomyoblastic tumor: A case report

**DOI:** 10.1097/MD.0000000000026105

**Published:** 2021-06-18

**Authors:** Yan Xia, Ye Li, Peng Gong, Huifeng Jiang, Xianbin Zhang

**Affiliations:** a Department of Pathology; b Department of Radiology, Qilu Hospital (Qingdao), Cheeloo College of Medicine, Shandong University, Qingdao, Shandong; c Department of General Surgery, Shenzhen University General Hospital & Carson International Cancer Research Centre, Shenzhen, P.R. China.

**Keywords:** histiocyte, histiocyte-rich, histiocyte-rich rhabdomyoblastic tumor, rhabdomyoblastic, skeletal muscle tumors

## Abstract

**Rationale:**

Skeletal muscle tumors are traditionally classified as rhabdomyomas or rhabdomyosarcomas. However, some soft tissue tumors cannot easily be identified as benign or malignant. We report a case of a histiocyte-rich rhabdomyoblastic tumor, with pathologic characteristics distinct from either rhabdomyoma or rhabdomyosarcoma. In contrast to rhabdomyosarcomas, the tumor cells exhibited low mitotic activity, lacking obvious morphologic atypia. Clinically, the tumor followed a very indolent course. Overall, the tumor did not fit classification criteria for either benign or malignant.

**Patient concerns:**

A 58-year-old Chinese man was admitted to Qilu Hospital on September 8, 2018, with a >20 year history of a mass in the middle of the left thigh. A few months prior to admission, he had experienced the pain from the mass extending to the distal left lower extremity. He had no prior history of significant disease or relevant family history.

**Diagnoses:**

Microscopically, numerous histiocytes and foamy cells covered the actual tumor cells that were positive for desmin, MyoD1, and myogenin, suggesting striated skeletal muscle cell differentiation. However, cross-striations were not detected in the tumor cells. The tumor was characterized by a non-infiltrative growth pattern and a low level of Ki67. A diagnosis of histiocyte-rich rhabdomyoblastic tumor was suggested.

**Interventions:**

The thigh mass was surgically resected September 12, 2018.

**Outcomes:**

The patient recovered well postoperatively, and was free of tumor recurrence or metastasis, followed to September 12, 2020 (23 months).

**Lessons:**

Histiocyte-rich rhabdomyoblastic tumor cells have minor atypia, indicating possible malignant potential. However, the tumor behavior was quit indolent. Due to the conflicting clinical and pathologic aspects of the tumor, to label it as rhabdomyosarcoma seemed inaccurate, potentially prompting over treatment. Interestingly, mutations were detected in *NF1*, *AXIN2*, *CHEK2*, *DNMT3A*, *KMT2D*, and *RB1* through next-generation sequencing. These mutations suggest disruptions in Ras signaling, the Wnt pathway, methyltransferases, and the cell cyclepotentially influencing the development of this histiocyte-rich rhabdomyoblastic tumor. This unusual tumor should be incorporated into the WHO Classification of Soft Tissue Tumors owing to its unique characteristics.

## 1 Introduction

The current WHO Classification of Soft Tissue Tumors divides skeletal muscle tumors into rhabdomyosarcomas and rhabdomyomas. Rhabdomyosarcoma is a malignant tumor^[1]^ typically affecting children and young adults^[2]^; it is a rapidly growing, aggressive tumor with necrosis and cytological atypia as the prominent morphologic features.^[3]^ Rhabdomyoma, a begin tumor,^[1]^ is reported in adults 20 to 87 years of age. It is characterized by non-infiltrative growth and mild behavior.^[4]^

In the present study, we report a histiocyte-rich rhabdomyoblastic tumor with pathologic and morphologic characteristics distinct from those of rhabdomyoma and rhabdomyosarcoma. Furthermore, we performed a next-generation sequencing analysis to screen genes that may be patholgenic for this tumor.

## 2 Case presentation

**Figure 1:**
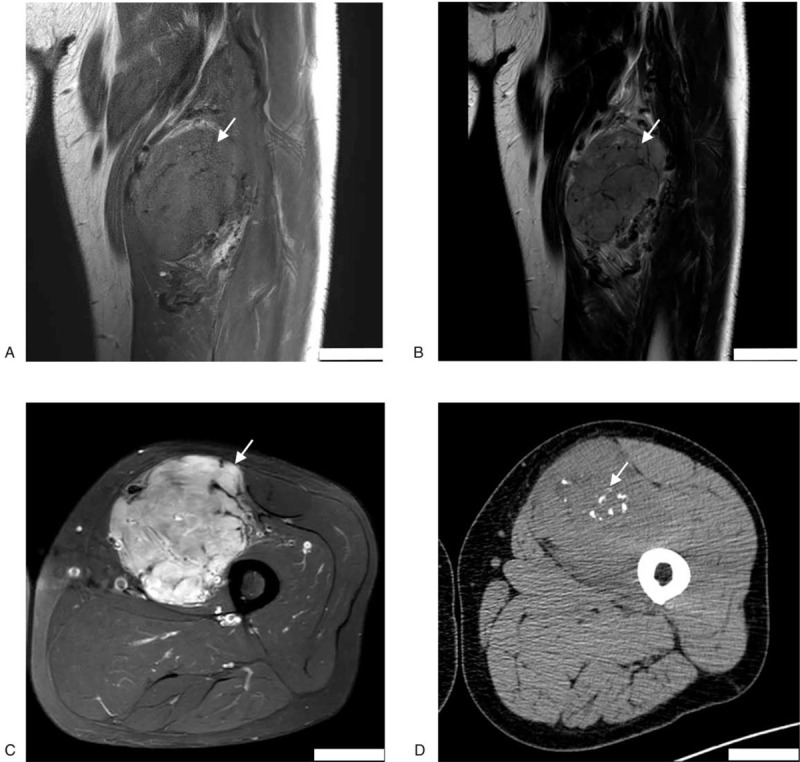
MRI and CT imaging of histiocyte-rich rhabdomyoblastic tumor. The T1-weighted (A), T2-weighted (B), MRI and CT (C) demonstrated a tumor in the left thigh. Several calcifications were observed in the tumor (D). CT = computed tomography, MRI = magnetic resonance imaging.

A 58-year-old Chinese man was admitted to the Qilu Hospital on September 8, 2018, with a >20 year history of a mass in the middle of the left thigh. A few months prior to admission, he had experienced pain from the mass extending to the distal left lower extremity. He had no prior history of significant disease or relevant family history. By magnetic resonance imaging (Fig. 1A and B) and computed tomography (CT) scan (Fig. 1C), the tumor (7.5 cm × 8.1 cm × 10.1 cm) was located among the muscles of the left thigh. In addition, several calcifications were apparent in the tumor (Fig. 1D). The patient underwent surgical resection of the tumor on September 12, 2018. The patient recovered well postoperatively and was free of tumor recurrence or metastasis, followed to September, 2020.

### 2.1 Gross pathology and microscopic examination

**Figure 2:**
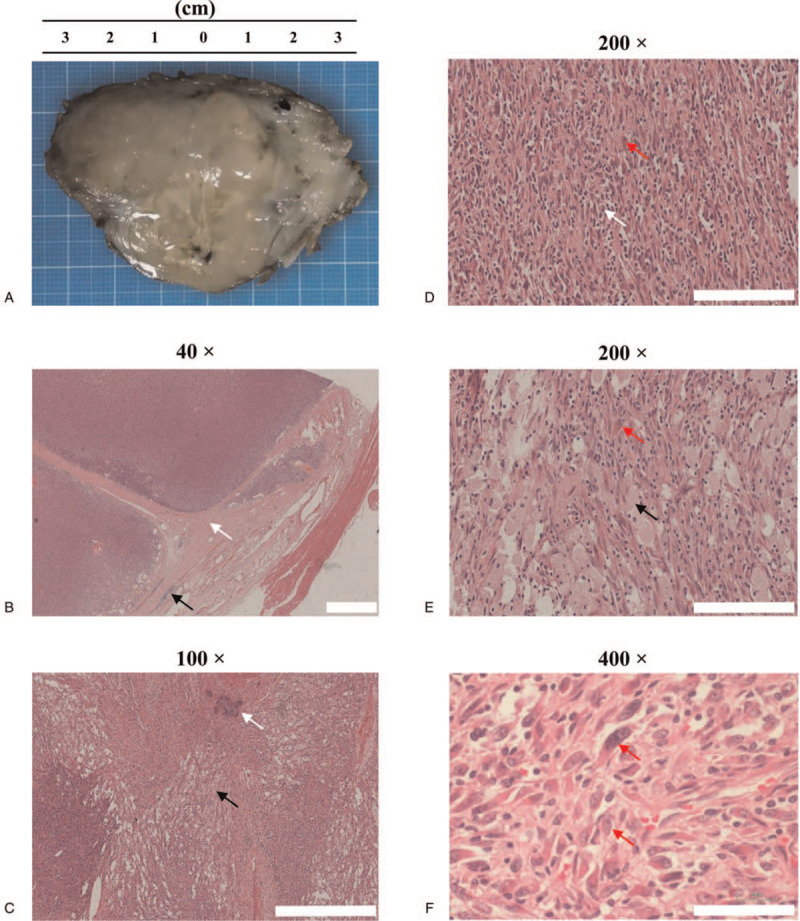
Pathological characters of histiocyte-rich rhabdomyoblastic tumor. The maximum diameter of this tumor was 9.0 cm (A), and this tumor was surrounded by fibrous tissues (B, indicated by the white arrow) and inflammatory cells (B, indicated by the black arrow). Consistent with the imaging analysis, several calcifications were observed under microscopy (C, indicated by the white arrow). The tumor was filled by amount of round or short spindle cells (D, indicated by the white arrow) and foamy macrophages (C and E, indicated by the black arrow). These cells hide the tumor cells behind them (D and E, indicated by the red arrow) and increase the false negative diagnosis of this tumor. The tumor cells have abundant eosinophilic cytoplasm and atypia of nuclei, large nuclei, prominent nucleolus and nuclear division (F, indicated by the red arrow).

The mass was a nodular tumor, maximum diameter of 9.0 cm with clear borders (Fig. 2A). Microscopically, the nodular tumor was surrounded by fibrous tissues (Fig. 2B, white arrow) and inflammatory cells (Fig. 2B, black arrow). Calcifications were present in the tumor (Fig. 2C, white arrow). These findings suggested the tumor might be benign. Surprisingly, when we observed the morphology under medium magnification (200×), we found that the tumor was filled with round or short spindle cells (Fig. 2D, white arrow). Notably, the boundaries between these cells were clear, and they had round or oval nuclei with rare cytoplasm. Moreover, we observed that numerous foamy macrophages (Fig. 1C and E, black arrows) also filled the tumor. These diffusely distributed round or short spindle cells and foamy macrophages hid the tumor cells, making the diagnosis difficult. In addition, we observed several long spindle cells or oval cells (Fig. 2D and E, red arrows), with abundant eosinophilic cytoplasm and nuclear atypia, including large nuclei, a prominent nucleolus, and nuclear division (Fig. 2F, red arrow). Interestingly, although the eosinophilic cytoplasm suggested these tumor cells could differentiate into skeletal muscle cells, we failed to observe the cross-striations that are typical characteristic of skeletal muscle cells.

### 2.2 Immunohistochemistry

**Figure 3:**
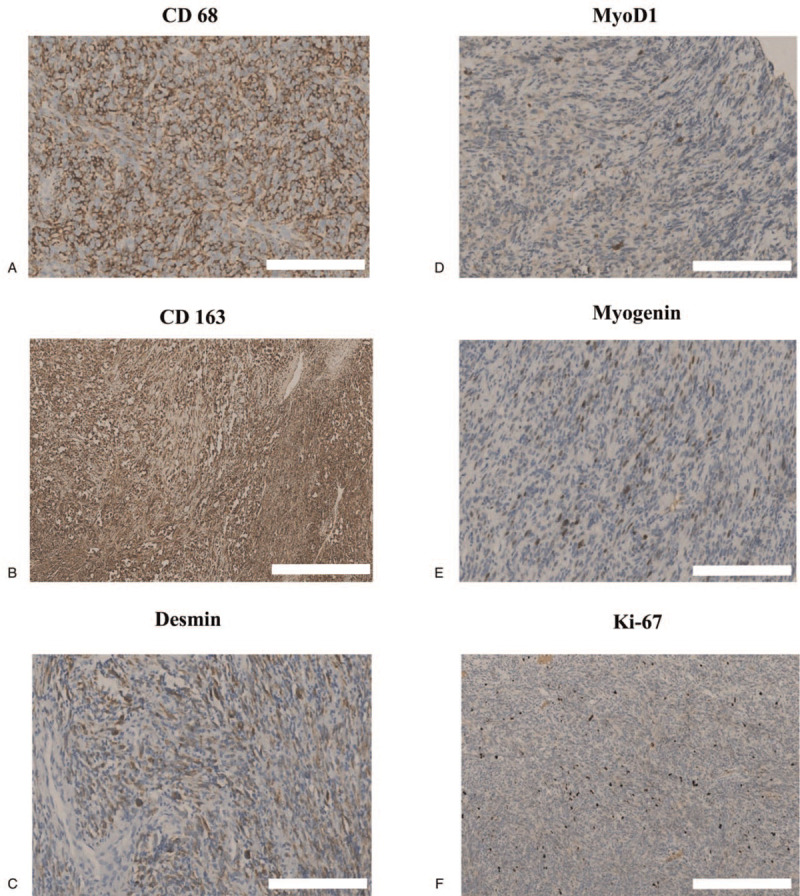
Immunohistochemistry assay of histiocyte-rich rhabdomyoblastic tumor. The tumor was stained by CD 68 (A) and CD163 (B). In addition, the positive of desmin (C), MyoD1 (D), and myogenin (E) suggested that these cells are rhabdomyoblastic cells. The Ki-67 index was only 5% in the hot spot area (F).

To determine whether these round or short spindle cells (Fig. 1D, white arrow) were histiocytes, the tissues were incubated with antibodies against CD68 and CD163, 2 histiocyte markers. We observed staining for both CD68 (Fig. 3A) and CD163 (Fig. 3B), establishing the cells as histiocytes. To investigate if the tumor cells were rhabdomyoblastic cells, the tissues were incubated with antibodies against desmin (Fig. 3C), MyoD1 (Fig. 3D), and myogenin (Fig. 3E). We observed that a few cells, hidden behind histiocytes and macrophages, were stained with these antibodies. The results provided strong proof the cells were indeed rhabdomyoblastic cells. To assess the proliferative activity of the tumor cells, Ki-67 staining was performed. While Ki-67-positive cells were rare, 5% were detected in the hot spot (Fig. 3F), indicating that the tumor had low proliferative activity.

### 2.3 Next-generation sequencing analysis

To identify potential causal mutations, a next-generation sequencing analysis was performed. Mutations were detected in *NF1*, *AXIN2*, *CHEK2*, *DNMT3A*, *KMT2D*, and *RB1* (Table 1).

**Table 1:** Next-generation sequencing analysis.

Gene	Mutation	
*NF1*	R304^∗^	exon 9
*AXIN2*	V457I	exon 6
*CHEK2*	A480T	exon 13
*DNMT3A*	Y724^∗^	exon 18
*KMT2D*	R4212Q	exon 39
*RB1*	R621S	exon 19

**Diagnosis:** Histiocyte-rich rhabdomyoblastic tumor.

## 3 Discussion

In 2019, Martinez et al^[1]^ reported that a small number of skeletal muscle tumors have pathologic characteristics and clinical behaviors distinct from rhabdomyosarcomas and rhabdomyomas. Specifically, these skeletal muscle tumors were literally filled with histiocytes, appropriately named “histiocyte-rich rhabdomyoblastic tumors of uncertain malignant potential.” Our findings indicated key differences between the pathological features of histiocyte-rich rhabdomyoblastic tumors and traditional skeletal muscle tumors. First, in contrast to rhabdomyosarcomas, histiocyte-rich rhabdomyoblastic tumor cells had low mitotic activity and lacked the usual obvious morphological atypia. Second, inflammatory cell infiltration and calcifications were observed in the present patient's tumor. Third, even with no treatment, the patient had survived for >20 years. These observations supported a benign classification for histiocyte-rich rhabdomyoblastic tumors. However, in comparison with benign skeletal muscle tumors, histiocyte-rich rhabdomyoblastic tumor cells have certain “malignant” morphologic characteristics, such as abundant eosinophilic cytoplasm, large nuclei, a prominent nucleolus, and nuclear division. These morphologic features suggest that histiocyte-rich rhabdomyoblastic tumors might have an intermediate behavior between rhabdomyosarcomas and rhabdomyomas.

The biological behavior of a tumor, benign or malignant, is crucial to determine treatment strategies. For example, the malignant behavior of rhabdomyosarcomas demands treatment with chemotherapy following surgical resection.^[5]^ Patients with rhabdomyoma, a benign tumor with innocent behavior, requires only local resection.^[6]^ Unfortunately, histiocyte-rich rhabdomyoblastic tumors cannot be precisely classified as either “benign” or “malignant,” and optimal treatment remains unclear.^[1]^

To identify potential mutations underlying the histiocyte-rich rhabdomyoblastic tumor, we performed next-generation sequencing. Consistent with previous results,^[1]^ we observed a mutation in *NF1*, a master regulator of the Ras signaling pathway,^[7]^*NF1* mutations have also been observed in rhabdomyosarcoma.^[8,9]^ Both rhabdomyosarcoma and histiocyte-rich rhabdomyoblastic tumors differentiate from skeletal muscle cells, and *NF1* regulates the growth and metabolism of muscle tissues.^[10]^ In addition, we observed mutations in *DNMT3A* and *KMT2D*, which encode methyltransferases,^[11]^ suggesting that methylation is involved in the development of histiocyte-rich rhabdomyoblastic tumors. Another pathophysiological mechanism of histiocyte-rich rhabdomyoblastic tumors might be the disruption of the cell cycle. This hypothesis is supported by the detection of mutations in *CHEK2* and *RB1*, that encode proteins and regulate the cell cycle.^[12]^ Additionally, we detected a mutation in *Axin2*,^[13]^ which encodes a scaffold protein in the Wnt signaling pathway, implying this pathway contributes to histiocyte-rich rhabdomyoblastic tumor development. Because of the rarity of this tumor, we have been unable to accumulate additional cases to achieve a more complete and definitive characterization of histiocyte-rich rhabdomyoblastic tumor. This is the limitation of our study.

## 4 Conclusion

Our findings have demonstrated that the morphology and behavior of histiocyte-rich rhabdomyoblastic tumors are distinct from those of traditional skeletal muscle tumors. Despite its atypical morphology, the clinical course of this tumor was quite indolent. This unusual tumor should be separately identified in the WHO Classification of Soft Tissue Tumors.

## Acknowledgments

This case was instructed by Professor Wangjian from the Department of Pathology of Fudan University Shanghai Cancer Center. The authors deeply appreciate the supports of Professor Wang and also give their sincerely gratitude to the medical staff from the Department of Pathology, Qilu Hospital, Shandong University.

## Author contributions

**Data curation:** Yan Xia, Ye Li.

**Investigation:** Yan Xia.

**Project administration:** Huifeng Jiang, Xianbin Zhang.

**Resources:** Peng Gong.

**Software:** Ye Li, Peng Gong.

**Supervision:** Huifeng Jiang, Xianbin Zhang.

**Writing – original draft:** Yan Xia.

**Writing – review & editing:** Xianbin Zhang.
